# Plaque geometry: determinant for fibrous cap stress levels

**DOI:** 10.1186/1532-429X-13-S1-P376

**Published:** 2011-02-02

**Authors:** Samuel A Thrysøe, Jens V Nygaard, Anders K Niemann, Nikolaj Eldrup, Anette Klærke, William P Paaske, Won Y Kim

**Affiliations:** 1 Aarhus University Hospital, Aarhus, Denmark; 2 Engineering College Aarhus, Aarhus, Denmark

## Introduction

In clinical practice, the risk of cerebrovascular events originating from carotid atherosclerotic plaques is correlated to the degree of luminal narrowing, commonly designated the degree of stenosis. Though the degree of stenosis is a proven marker of plaque vulnerability, it is widely recognized that better risk markers for cerebrovascular events are needed. Known morphological features of plaque vulnerability are large lipid-rich necrotic cores (LR-NC) with thin fibrous caps that generate localized elevated mechanical stresses within the fibrous cap separating arterial lumen from LR-NC. Thus, determination of local biomechanics using computational simulations may have clinical implications.

## Objective

Biomechanical stress levels could be indicative of plaque rupture risk. Thus, we wished to determine whether different plaque morphologies in a longitudinal section affects the level of local mechanical forces.

## Methods

**Figure 1:**
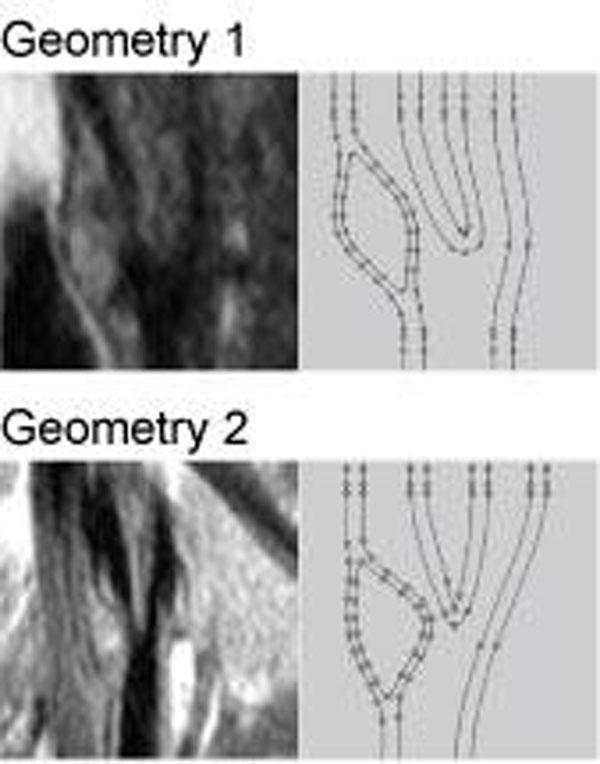
The two patient geometries simulated. Left: MRI scan, right: Computational model

Two patients scheduled for carotid endarterectomy were scanned using MRI and idealized anatomical 2D models of individual carotid bifurcations were created (figure 1). Keeping the outer vessel wall constant, 3654 simulations were performed with varying morphological parameters in the form of patient geometry, fibrous cap thickness, LR-NC size, degree of stenosis, and blood pressure. The inner vessel wall and LR-NC boundaries were adjusted as needed to satisfy the requested morphology. The models were simulated employing fluid structure interaction analyses using COMSOL multiphysics 4.0a and analyzed with Matlab R2010a. Mechanical stresses were evaluated using a widely adopted critical threshold of 300 kPa below which the plaque is not considered at risk of rupture.

## Results

**Figure 2:**
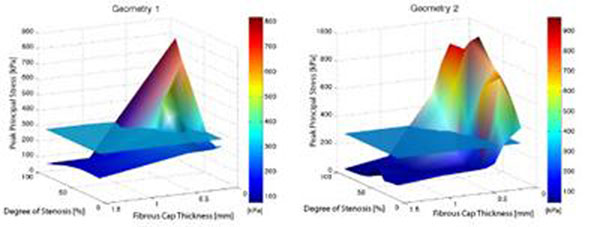
Peak principal stresses as a function of the degree of stenosis and fibrous cap thickness. Blood pressure was 160 mmHg in all the simulations. The simulations were performed using identical morphological parameters, with only the patient geometry varying. This resulted in remarkably different profiles, particularly at low fibrous cap thickness.

**Figure 3:**
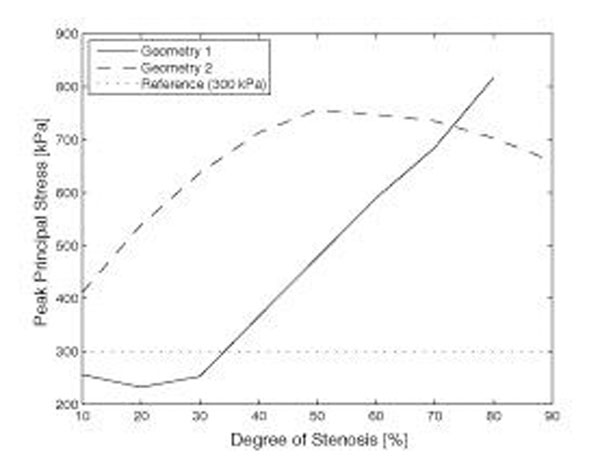
Peak principal stresses as a function of the degree of stenosis. All simulations were performed using a fixed fibrous cap thickness of 0.1mm and at 160 mmHg blood pressure.

**Figure 4:**
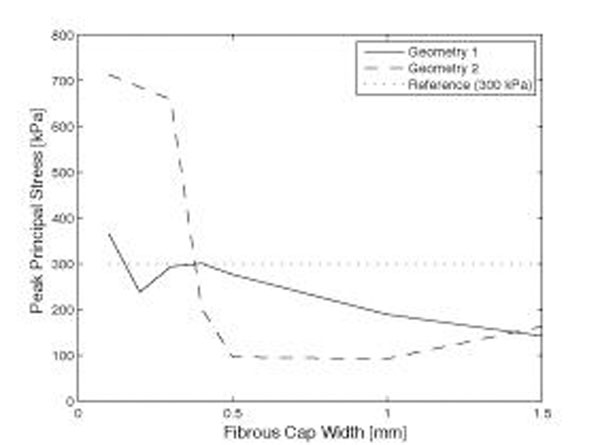
Peak principal stresses as a function of the degree of stenosis. All simulations were performed using a fixed degree of stenosis of 70% and at 160 mmHg blood pressure.

Significant differences were apparent comparing the two different patient morphologies using surface plots of degree of stenosis, peak principal mechanical stresses, and fibrous cap thickness (figure 2). In particular, a fibrous cap thickness of 0.1 mm yielded stresses above 300 kPa in geometry 2 regardless of the degree of stenosis, while geometry 1 remained below the threshold for lower degrees of stenosis (figure 3). Plotting peak principal stresses as a function of diminishing fibrous cap thickness (employing a constant degree of stenosis of 70%), the differences between the two geometries were pronounced with a fibrous cap thickness of less than approximately 0.3 mm (figure 4).

## Conclusions

Identical plaque morphologies may yield significantly different mechanical stress levels depending on vessel geometry. Further studies are needed to determine if the varying stress levels are indicative of differing risks of plaque rupture.

